# Overcoming Barriers to Women's Career Transitions: A Systematic Review of Social Support Types and Providers

**DOI:** 10.3389/fpsyg.2022.777110

**Published:** 2022-05-26

**Authors:** Tomika W. Greer, Autumn F. Kirk

**Affiliations:** Department of Human Development and Consumer Sciences, College of Technology, University of Houston, Houston, TX, United States

**Keywords:** career transition, career development, women, social support, literature review, support network

## Abstract

In the current career landscape and labor market, career transitions have become a critical aspect of career development and are significant for Human Resource Development (HRD) research and practice. Our research examines the type of support used during different career transitions and who can provide that support to women in career transition. We investigated four types of social support—emotional, appraisal, informational, and instrumental—and their roles in five types of career transitions: school-to-work transition, upward mobility transition, transition to a new profession, transition to entrepreneurship, career re-entry transition, and transition to retirement. We analyzed 80 journal articles using directed content analysis, cross-tabulation, and nonparametric statistical tests. Instrumental support appears to be the most commonly documented type of social support in this career transition literature. Appraisal support was consistently documented least for each type of career transition. Our results may highlight the importance of personal connections and internal resources in successful career transitions for women. Based on our findings, we offer a model of women's social support network for career transitions and advocate for expanded networks of social support for women anticipating and during career transitions. The results of our study contain insights for how women can be supported in transitioning to the next career experience. We conclude with suggestions for future research.

## Introduction

A career transition refers to “the period during which an individual adjusts to a different setting and/or work role” (Louis, 1982, p. 73). In the current career landscape and labor market, career transitions have become a critical aspect of career development as individuals more frequently cross “the boundaries of occupations, industries, organizations, functional areas, countries, and the labor market” (Sullivan and Al Ariss, 2021, p. 1). Likewise, Sun and Wang (2009) suggested that career transitions are significant for Human Resource Development (HRD) research and practice.

In their review of literature on career transitions, Sullivan and Al Ariss (2021) noted the need for more research related to the quality and type of support offered by members of an individual's social network. Given the social embeddedness of career transitions, relationships with people in the work domain and the non-work domain can influence career transition decisions and how individuals adjust to career transitions. Though social support is known as an important resource before, during, and after career transitions, little is known about which types of support are received from different members of one's social network (Sullivan and Al Ariss, 2021).

Career transition experiences may differ based on the type of support a transitioner receives from multiple sources (Malecki and Demaray, 2003). Several types of support can facilitate successful career transitions. These different types of support should be studied and recognized for their role in helping individuals through career transitions (Barling et al., 1988; Malecki and Demaray, 2003). Likewise, an understanding of who provides social support has implications for the practical implementation of offering support for career transitions. Social support for career transitions can come from workplace relationships, including coworkers and supervisors, and from nonwork relationships, including spouses/partners, family, and friends (Baruch-Feldman et al., 2002; Halbesleben, 2006; Van Daalen et al., 2006; Alsubaie et al., 2019). Furthermore, the impact of social support on career transitions is mediated by self-efficacy and other self-management support (Wang and Fu, 2015; Hou et al., 2019). We, therefore, view the *self* as an important contributor to the relationship between social support and career transitions. Our research examines the type of support used in different career transitions and who can provide that support to women in transition. Due to the mediating role of self-initiated factors, we also acknowledge that the women, themselves, are contributors to the total impact of social support on successful career transitions.

In this study, we were guided by House's (1981, p. 22) question “*Who* gives *what* to *whom* regarding *which* problems?” As it relates to social support during women's career transitions, we have adopted House's question as follows: *Who* gives which types of *social support* to women during different types of *career transitions*?

## Social Support and Career Transitions

Social support has long been considered a notable contributor to the career transition process. Kahn (1976) recognized the role of social support in helping people manage the changes in their major social roles. Subsequently, House's (1981) conceptualized four types of support that could improve individual effectiveness and reduce work-related stress: emotional support, appraisal support, informational support, and instrumental support. Social support was later applied specifically to career transitions because social support can serve as a coping resource for adults in transition to adapt to life changes (Schlossberg, 1981; Sargent and Schlossberg, 1988). Coping resources impact the strategies used to navigate the career transition (Thoits, 1995). When social support is not perceived during times of change and transition, people may experience feelings of isolation, anxiety, or develop negative coping strategies (Clowes et al., 2015; Byrne and Theakston, 2016; Buhl et al., 2018).

Emotional support is primarily affective and includes empathy, caring, love, and trust (House's, 1981). Emotional support includes building workplace relationships, mentoring, self-efficacy, or support from family and friends (Colbert et al., 2016; Buhl et al., 2018). In contrast, instrumental support offers tangible help to recipients in need (House's, 1981). Instrumental support can include providing someone with materials or resources, such as money or time (Malecki and Demaray, 2003). Informational support involves only the transmission of information, advice, directives, guidance, or suggestions from the support provider to the recipient (House's, 1981). Appraisal support offers evaluative feedback for the recipient (Tardy, 1985). The evaluative feedback associated with appraisal support can be implicitly evaluative (e.g., not correcting or redirecting someone's actions) or explicitly evaluative (e.g., telling someone they did a good job) (House's, 1981).

Individuals may rely on all four types of social support as important coping resources for navigating career transitions across the span of their careers. Women's contemporary careers offer a rich context for studying career transitions because of the myriad of choices that women make as they move in and out of full-time work, part-time work, and career breaks in response to their evolving needs for authenticity, balance, and challenge (Zimmerman and Clark, 2016). Compared to men, women's careers are less likely to be linear due to pressures and social norms that imply women should be primarily responsible for caretaking and domestic responsibilities in the home, which strains their capacity to formulate an uninterrupted progressive career (Cabrera, 2009; Cross, 2010; Kuitto et al., 2019; Maxwell et al., 2019). For example, globally, 30% of young women are not in employment, education, or training; compared to just 13% of men who are not in employment, education, or training (ILO, 2019). This gap in workforce participation suggests that, compared to men, women are disadvantaged in their access to career development opportunities, including career transitions. Additionally, women's careers are more often influenced by their social interactions and personal relationships (Conlon, 2004), which allows social support (or lack of) to impact their career transition experiences. In our study, we considered five career transitions that women may encounter – all of which present potential barriers, or problems, that may be reduced by receiving social support.

## Barriers to Women's Career Transitions

Career trajectory refers to the entire timeline of one's career from beginning to end (Bandura et al., 2001). Career trajectories are complex patterns marked by variables such as gender, ethnicity, socioeconomic background, age or self-efficacy (Bandura et al., 2001; Bimrose et al., 2013; Gubler et al., 2015). The intersectionality of these variables, and others, determine the career choices women make and the opportunities they have throughout their career trajectories (Johnston et al., 2015; Atherwood and Sparks, 2019).

### School-to-Work Transition

The school-to-work transition encompasses any transition from formal education into the workforce. Traditionally, this involves students transitioning from higher education and “university life” to the career path they chose for themselves and studied for throughout college. However, this career transition category may also include the transition from high school, technical or trade school, or other types of education typically required of or pursued by young adults as they prepare themselves to enter the workforce (Murphy et al., 2010; Maxwell and Broadbridge, 2014).

School-to-work transitions can be stressful and difficult for those making the transition. As showcased by Mills (2017), young people have struggled with the transition from school to work for decades and continue to do so as this transition typically accompanies the broader transition from childhood into adulthood (Murphy et al., 2010). More recently, researchers have reported that many postgraduate students, particularly from rural areas or low-income backgrounds, felt their formal education was insufficient, varied in quality, and lacked career guidance, leaving graduates feeling markedly unprepared to enter the workforce (Eley, 2010; Hamilton, 2019). This transition, as with many others, is made increasingly more complicated when students are navigating it with a significant other (Domene et al., 2012). Additionally, students who choose to leave school early often experience higher unemployment, lower paying jobs, and may be subject to a higher risk of exclusion from their peers (Van Praag and Clycq, 2020).

These challenges during the school-to-work transition are amplified by “issues of access, debt, and contingent employment” (Martin and Frenette, 2017, p. 2). Women, specifically, have cited barriers in the school-to-work transition such as balancing career and family while also trying to live up to the expectation that they can and should have it all (Seminario, 2018). Other barriers frequently described for women in the school-to-work transition included lack of female role models and mentors, maternal profiling, and penalties imposed on one's career for taking time to marry and/or raise families (Eley, 2010; Domene et al., 2012; Grant, 2012; Bieri et al., 2016; Sassler et al., 2017; Lindsay et al., 2019).

### Upward Mobility Transition

The upward mobility career transition involves moving into a higher-ranking position within a specific profession. This type of transition often entails a woman acquiring training, education, or experience in the lower position before becoming eligible to move into the higher position. One of the most notable barriers that plagues upward mobility transitions is the “gender gap”, a term used to describe the underrepresentation of women in certain professions, industries, and organizational leadership (Cross, 2010; Arar, 2014; Amon, 2017; Lerchenmueller and Sorenson, 2018). Other barriers include lack of available desirable positions (Rybarczyk et al., 2016), not learning about new responsibilities (Westerman et al., 2013), struggling to build a new identity and confidence within the new job role (Schor et al., 2011; Arar and Shapira, 2012; Amon, 2017), and difficulties finding positive role models and mentors to help guide the individual through the career transition (Shaw and Stanton, 2012).

### Transition to a New Profession

A career change to a new profession could be defined as completely moving out of the current career trajectory and beginning a new career path. This type of career transition may commonly be characterized by a return to university or other type of education or training to bolster skills acquired in prior career or to acquire new skills. The new profession may be related to the prior profession, such as leaving a career as a professional athlete to begin a career as a coach (Park et al., 2012). Other times, the new profession may be less obviously related, such as leaving a career in the military to join the civilian workforce (Greer, 2017).

This type of career transition can be particularly challenging when the transition is not voluntary. If a woman was terminated from her previous job and chooses to seek employment in a new industry or profession, she may harbor negative feelings about leaving her prior career field (Snyder et al., 2013; Byrne and Theakston, 2016; Yakaboski, 2016). Often, women transitioning into a completely new field experience difficulties as they re-learn how to navigate the job market or when they are over- or underqualified for positions in new industries. These difficulties can lead to decreases in self-efficacy, loss of identity, or feelings of failure, anxiety, and low self-esteem (Snyder, 2011; Park et al., 2012; Tshube and Feltz, 2015; Byrne and Theakston, 2016; Bergman and Herd, 2017; Pellegrino et al., 2018). A transition to a new profession may also be accompanied with lifestyle changes that affect the woman's whole family, such as relocating, reduction in income, or working greater or fewer hours than is optimal for the well-being of the whole family (Yakaboski, 2016; Greer, 2017; McMahon et al., 2018).

### Transition to Entrepreneurship

Many women choose to pursue an entrepreneurial career to help balance parenthood and work (Duberley and Carrigan, 2013; Lewis et al., 2015). Women also choose to pursue entrepreneurial careers to achieve more financial freedom and control over how they spend their time (Patterson and Mavin, 2009; Hodges, 2012). However, many entrepreneurs expressed a feeling of disillusionment after realizing they made less money and worked longer hours in the beginning stages of self-employment (Hodges, 2012; Duberley and Carrigan, 2013). New entrepreneurs must gain credibility among their peers and confidence in themselves as they explain their decisions to their peers, while often feeling like others perceive them as unemployed (Patterson and Mavin, 2009). Additionally, many new entrepreneurs have trouble separating home and work life, which amplifies stress about being a successful entrepreneur and a successful parent (Lewis et al., 2015).

### Career Re-entry Transition

The career re-entry transition occurs when someone has taken time away from their career and has chosen to re-enter their career in a similar job and position as to when they left. Many women experience career re-entry following childbirth and maternity leave (Fehring and Herring, 2012; França, 2012; Arntz et al., 2017; Hennekam et al., 2019). While transitioning back into the workforce, they may feel pressured to return to work as soon as possible and feel guilty for not continuing their leave to spend time with their child(ren) during the most formative years (Arntz et al., 2017). Finding work-life balance may also be especially difficult for women who have begun to redefine their aspirations and priorities while away from work (Cabrera, 2009). Many women feel their job role is redundant once they return to work, as other employees may have been hired to fill their position while they were away from work (Cameron, 2009). This situation can create additional concerns about financial stability and job security.

Longer periods of leave, such as career re-entry after several years of caring for a child or incarceration, can create even bigger challenges. Returners may feel disconnected from the work world and may struggle to re-learn skills and regain confidence (Cameron, 2009; Greer, 2013; Heidemeier and Wiese, 2014). Additionally, taking a break from one's career may penalize women returners by only allowing them to re-enter at a lower position or lower income (Zimmerman and Clark, 2016).

### Transition to Retirement

The retirement transition signifies the end of one's career trajectory. Often, during the retirement transition, people take time to discover their interests, spend time with their families, travel, or pursue their hobbies. This transition may be particularly challenging for individuals retiring before they are financially ready or if they are experiencing health declines that prevent them from working anymore (Byles et al., 2013). When retiring, some women feel a loss of their identity and their workplace friends, which may cause negative feelings about retirement (Myers, 2011; Clowes et al., 2015; Duberley and Carmichael, 2016). The goal of many retirees is to maintain their lifestyle into retirement but, for some, this is not possible and may increase anxiety about whether they can live self-sufficiently in retirement (Duberley and Carmichael, 2016). These challenges can cause feelings of isolation, shame, and fear that lead to psychological withdrawal from life after work (Park et al., 2012).

## Method

We conducted a systematic review of literature to identify the types of social support that women can receive in order to overcome the barriers and problems associated with these five different career transitions. We searched five electronic databases (Academic Search Complete, Business Source Complete, ERIC (Ebscohost), PsycARTICLES, and PsycINFO) for relevant peer-reviewed journal articles published between 2009 and 2019. We chose a ten-year time frame to bound the search results to a manageable number of articles. This time frame also provided insight into the contemporary career landscape as economies recovered from the 2008 economic recession. The literature search was conducted at the beginning of 2020.

We identified 479 search results that used the keywords “career transition” and “women/woman/female/females” in the article abstract. We manually screened these 479 articles and selected the articles that were most relevant to this study. Upon initial review, we eliminated articles related to transitions into university, transitions from secondary to tertiary education, sexual transitions, transitions in location, such as expatriation or repatriation, and any articles whose central purpose was not to describe career transitions. Second, we eliminated books, theses/dissertations, and conference papers. After removing duplicates from the resulting list, we retained 80 journal articles for analysis in our literature review. The final selection included 47 qualitative studies, 23 quantitative studies, 5 studies containing mixed methods, and 5 conceptual studies. This variety of methodologies offered an opportunity for us to investigate a wide range of types and sources of support through people's actual experiences, through researchers' perceptions of others' experiences, and through hypotheses testing.

Our sample is slightly more robust than other recent reviews of the career transition literature. For example, de Vos et al. (2021) analyzed 34 articles to assess relationships between career transitions and employability and Guan et al. (2019) included 61 articles in their review of the relationships between career transitions and career success. We extended our review beyond the scope of de Vos et al. (2021) and Guan et al. (2019) to include school-to-work transitions and retirement transitions. Though we are unlikely to identify every article in existence that could be included in this review, we aimed to review enough articles to find any salient trends in the career transition literature. In sum, we identified and analyzed 32 articles related to the school-to-work transition, 12 articles related to upward mobility transitions, 13 articles related to career transitions to a new profession, five articles related to career transitions to entrepreneurship, nine articles related to career re-entry, and nine articles related to transitions to retirement.

### Data Analysis

We used a combination of qualitative and quantitative approaches to analyze our data. First, we performed a directed content analysis (see Hsieh and Shannon, 2009) of the articles to identify who provided what types of social support to women during career transitions. The directed content analysis was used because we were specifically looking to describe the manifestation of four types of social support as defined by (House's, 1981) framework. We, therefore, approached this phase of the content analysis with pre-determined coding categories for social support. Second, we used Chi-square Tests of Independence and pairwise Z-Tests to investigate patterns in the data regarding who was providing which types of support for women during different types of career transitions. Our quantitative data analysis helped to determine if there were significant associations between type of transition, type of support, and who provided the support.

## Results

The first phase of data analysis allowed us to determine that across the six types of career transitions, there were eight sources of social support for women during their career transitions. As shown in Supplementary Table 1, social support could come from the organization/employer, family and friends, peers and colleagues, mentors, supervisors, educators and trainers, childcare providers. Additionally, women, themselves, played a notable role in providing support for their own career transitions. For example, women who were able to maintain intrinsic motivation and persistence were more successful in obtaining social support (Damaske, 2009; Lörz and Mühleck, 2019). Similarly, women who communicated clearly and openly with social supporters and actively participated in self-reflective exercises with the aid of their supporters experienced more successful transitions (Doerschuk et al., 2016; Hartung and Vess, 2016; Finn, 2017). In other words, women who actively sought out and participated in support were more successful in their career transitions. In Supplementary Table 1, we have included a description of the documented social support to illustrate what practical support can be given or received to enable a career transition. This qualitative data provided answers for our question of who can give what support during a career transition. In reporting our results, we acknowledge the results are based on which sources and types of support were documented in the literature. In very few cases were all types of support examined within a single article.

To elucidate the potential nuances in this data, we quantified the data to look for any patterns in explaining who was assigned to what support during a career transition. Table 1 contains the summarized data produced from a cross-tabulation, representing the total number of identified instances of social support attributed to each provider category. In total, we coded 310 instances of social support identified in our literature sample. Overall, instrumental support (*N* = 147) appears to be the most commonly documented type of social support in this career transition literature. Appraisal support (*N* = 27) was consistently documented least overall and for each type of career transition.

**Table 1:** Summary of sources and types of support for all transition types.

**Type of transition**	**Source of support**	**Emotional support**	**Informational support**	**Instrumental support**	**Appraisal support**	**Total**
School-to-work transition	Self	3	4	31	6	**44 (36%)**
	Organization/employer	1	6	10	0	**17 (14%)**
	Family and friends	20	0	7	0	**27 (22%)**
	Peers and colleagues	1	0	3	0	**4 (3%)**
	Mentors	0	0	8	0	**8 (7%)**
	Supervisors	0	0	0	0	**0 (0%)**
	Educators and trainers	0	18	2	0	**20 (17%)**
	Childcare providers	0	0	1	0	**1 (1%)**
	**Total**	**25 (21%)**	**28 (23%)**	**62 (51%)**	**6 (5%)**	**121**
Upward mobility transition	Self	0	1	13	1	**15 (33%)**
	Organization/employer	0	0	3	1	**4 (9%)**
	Family and friends	6	0	0	0	**6 (13%)**
	Peers and colleagues	0	0	3	0	**3 (7%)**
	Mentors	0	0	6	0	**6 (13%)**
	Supervisors	0	0	0	3	**3 (7%)**
	Educators and trainers	0	7	1	0	**8 (18%)**
	Childcare providers	0	0	0	0	**0 (0%)**
	**Total**	**6 (13%)**	**8 (18%)**	**26 (58%)**	**5 (11%)**	**45**
Career transition to new	Self	6	3	6	5	**20 (37%)**
profession	Organization/employer	2	0	5	0	**7 (13%)**
	Family and friends	8	0	1	0	**9 (17%)**
	Peers and colleagues	2	1	2	0	**5 (9%)**
	Mentors	2	0	1	0	**3 (6%)**
	Supervisors	0	0	0	0	**0 (0%)**
	Educators and trainers	0	9	1	0	**10 (19%)**
	Childcare providers	0	0	0	0	**0 (0%)**
	**Total**	**20 (37%)**	**13 (24%)**	**16 (30%)**	**5 (9%)**	**54**
Career transition to	Self	3	0	4	3	**10 (45%)**
entrepreneurship	Organization/employer	0	0	0	0	**0 (0%)**
	Family and friends	2	0	3	0	**5 (23%)**
	Peers and colleagues	0	0	0	0	**0 (0%)**
	Mentors	1	1	1	0	**3 (14%)**
	Supervisors	0	0	0	0	**0 (0%)**
	Educators and trainers	0	2	0	0	**2 (9%)**
	Childcare providers	0	0	2	0	**2 (9%)**
	**Total**	**6 (27%)**	**3 (14%)**	**10 (45%)**	**3 (14%)**	**22**
Career re-entry transition	Self	3	1	9	2	**15 (45%)**
	Organization/employer	2	0	8	0	**10 (30%)**
	Family and friends	4	0	0	0	**4 (12%)**
	Peers and colleagues	0	0	0	0	**0 (0%)**
	Mentors	0	0	1	0	**1 (3%)**
	Supervisors	0	0	0	0	**0 (0%)**
	Educators and trainers	0	1	0	0	**1 (3%)**
	Childcare providers	0	0	2	0	**2 (6%)**
	**Total**	**9 (27%)**	**2 (6%)**	**20 (61%)**	**2 (6%)**	**33**
Retirement transition	Self	3	0	4	6	**13 (37%)**
	Organization/employer	0	0	9	0	**9 (26%)**
	Family and friends	10	0	0	0	**10 (29%)**
	Peers and colleagues	0	0	0	0	**0 (0%)**
	Mentors	0	0	0	0	**0 (0%)**
	Supervisors	0	0	0	0	**0 (0%)**
	Educators and trainers	0	3	0	0	**3 (9%)**
	Childcare providers	0	0	0	0	**0 (0%)**
	**Total**	**13 (37%)**	**3 (9%)**	**13 (37%)**	**6 (17%)**	**35**
**Grand totals**		**79**	**57**	**147**	**27**	**310**

During a transition into a new profession, emotional support was more frequently noted, representing 37% of the documented support for this type of transition. Most of this emotional support was attributed to family and friends (40%) and the woman, herself (30%). During a transition into an entrepreneurial career, instrumental support was most commonly noted, amounting to 45% of the documented support for the transition to entrepreneurship. This instrumental support was primarily assigned to the *self* (40%) and family and friends (30%). Career re-entry transitions were also primarily sustained by instrumental support, which represented 61% of all documented support for career re-entry transitions. For career re-entries, the instrumental support was credited to self-support (45%) and support from the organization/employer (40%). For the retirement transition, emotional support and instrumental support were recognized equally—together, amounting to 74% of all documented support for retirement transitions. Most (77%) of the emotional support was attributed to family and friends, while the instrumental support was primarily (69%) attributed to the organization/employer during the retirement transition. The school-to-work transition had the most instances of social support coded in this data set. Notably, instrumental support was most frequently identified, accounting for 51% of the social support documented for the school-to-work transition. More than 50% of this instrumental support was credited to the *self*. Instrumental support was also the most frequently (58%) coded support type for the upward mobility transition. During the upward mobility transition, 50% of the instrument support was attributed to the *self*.

A Chi-Square Test of Independence was used to determine whether there were significant associations between type of career transition and the type of social support. There was a significant relationship between the type of career transition and the support type [χ^2^(15, 310) = 28.76, *p* = 0.017], suggesting a detectable pattern in this dataset that indicates different types of support were associated with different types of career transitions. A second Chi-Square Test of Independence was used to detect any significant associations between source of support and the type of social support. There was a significant relationship between the source of support and the support type [χ^2^(21, 310) = 353.05, *p* <0.001], offering some evidence of statistical differences in the frequencies of supporters contributing different types of social support to career transitioners.

**Figure 1:**
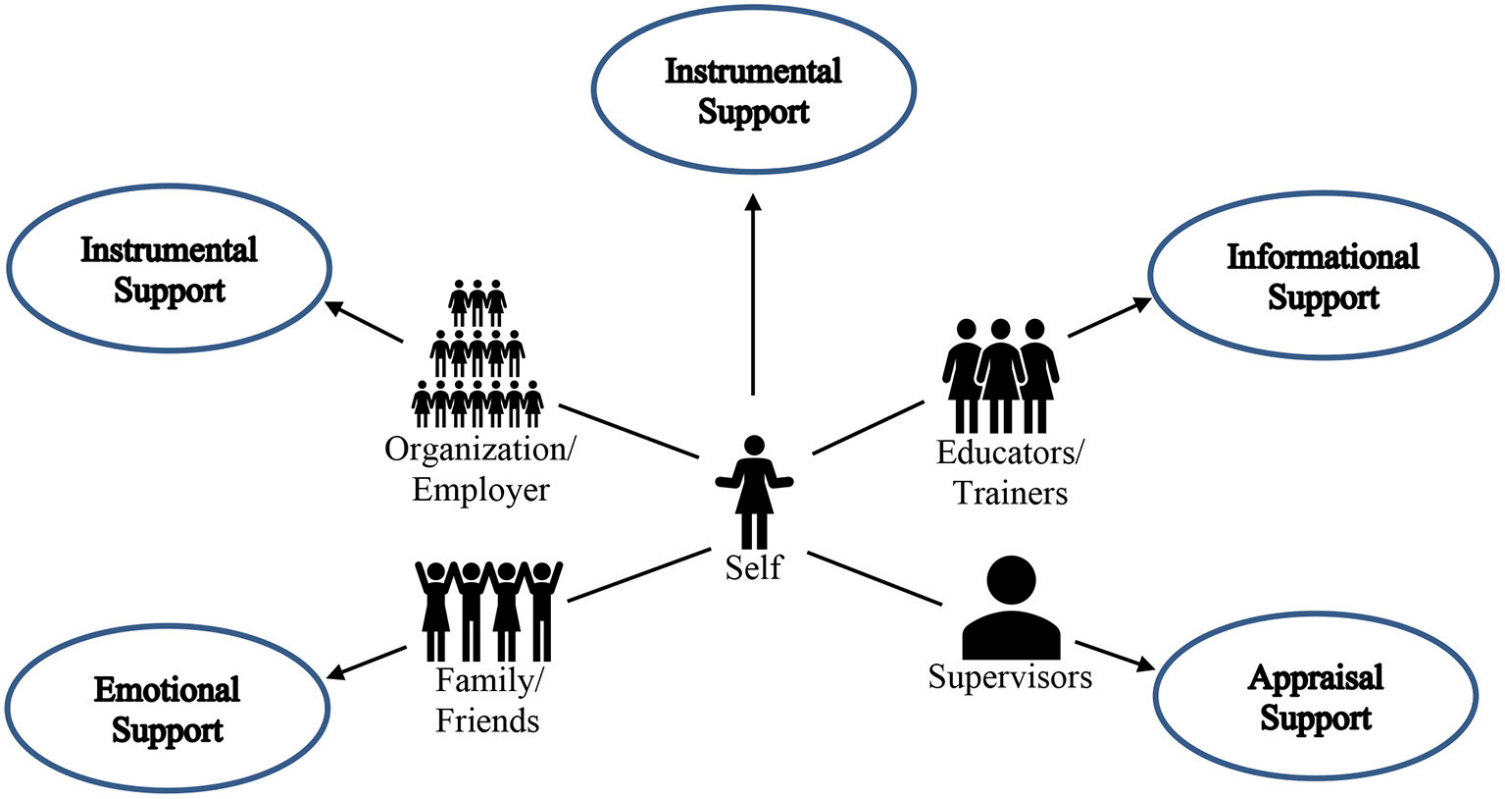
Women's social support network for career transitions.

Finally, *post hoc* Z-Tests with a Bonferroni correction were used to determine which proportions in the dataset were significantly different. Here, we summarize the significant relationships. For career transitions to new professions, emotional support was reported significantly (*p* < 0.05) more than instrumental support. There were no other significant differences related to the type of career transition and the type of support. Comparing sources of support and types of social support (see Figure 1), instrumental support was documented significantly (*p* < 0.05) more than emotional support from organizations/employers. Similarly, instrumental support was documented significantly (*p* < 0.05) more than all other support from the *self*. Emotional support was documented significantly (*p* < 0.05) more than all other types of support from family/friends. Appraisal support was documented significantly (*p* < 0.05) more than emotional support and instrumental support from supervisors. Informational support was documented significantly (*p* < 0.05) more than all other support from educators/trainers.

## Discussion

In our research, we investigated social support as a coping resource for overcoming barriers to women's career transitions (Thoits, 1995). We examined the extant literature to determine which types of social support have been documented and who is credited with the support for women in career transitions. Our approach utilized qualitative data analysis and quantitative data analysis to provide insights on this phenomenon. The findings support (House's, 1981) conceptualization of social support as we found evidence of all four types of support—emotional, informational, instrumental, and appraisal—in our review of 80 journal articles.

### Theoretical Contributions

Our findings align with previous research that concluded different types of social support can have differential effects on stressful situations (e.g., Barling et al., 1988). The results of our Chi-Square tests reinforce the idea that there are statistical differences in the frequencies of support types documented for different types of career transitions. While we found evidence in the extant literature that all four types of support could potentially contribute to a variety of career transitions for women, the results of our analyses suggest that different support types are documented more frequently for some career transitions rather than equally distributed across all types of career transitions. Specifically, our data suggest that emotional support may be a more common resource for effective transitions to a new profession than instrumental support. The detected differences among support types for different types of career transitions can inform theoretical understanding of the relationship between types of social support and types of career transitions. Aligning the types of social support with the types of career transitions allows for a nuanced theoretical approach to explaining the factors that contribute to successful career transitions.

As observed by Sullivan and Al Ariss (2021), little is known about the type of social support provided by different members of one's social network during a career transition. In our review of the literature, we identified eight sources of social support: *self*, organization/employer, family and friends, peers and colleagues, mentors, supervisors, educators and trainers, and childcare providers. The literature contains evidence that each of these providers of social support can contribute to successful career transitions for women. The results of our Chi-Square and Z-tests suggest statistical differences in the frequencies of support types attributed to different support providers across the literature we reviewed. While we found evidence in the extant literature that support providers in the eight categories could potentially provide different types of social support for women during career transitions, the results of our analyses suggest that different support types are attributed more frequently to some support providers rather than equally distributed across all eight categories of support providers. Based on the findings of our study, we constructed a model of women's social support network for career transitions (Figure 1). By identifying these sources of support, we offer a basis on which to advance understanding of the role that specific support providers in one's social network can play in helping the individual successfully navigate a career transition. A diverse network of social support providers can give women access to the full range of resources, information, career development, and psychosocial support needed to make a successful career transition (Greer and Minnis, 2022). Therefore, expanded networks of social support for women anticipating and during career transitions can contribute to successful transitions.

Though we recognize the importance of social support networks that include people inside and outside the workplace, our results also highlight the importance of the *self* as a source of emotional, informational, instrumental, and appraisal support to aid in successful career transitions. Our *post hoc* analyses suggested that instrumental support is more commonly documented from the *self* while emotional and informational support are significantly less documented. Our findings are consistent with previous research that demonstrated the importance of internal resources (self-esteem and mastery) compared to external social support. Bovier et al. (2004) found internal resources to be directly beneficial to health outcomes, while social support had an indirect effect on the desired outcomes. Similarly, internal resources (e.g., self-efficacy) have previously been noted as critical mediators and moderators of the relationships between social support and career outcomes (Wang and Fu, 2015; Hou et al., 2019). Our study contributes to increased understanding and identification of specific internal resources that can contribute to the social support and career transitions models.

### Practical Implications

#### Social Support and Career Transition Types

Based on our review of the literature and the proportions calculated in the resultant dataset (see Table 1), support providers can emphasize emotional support for women moving into new professions. Strong emotional ties with members of her social network help to create the developmental relationships that propel a career change to a different profession (Greer and Minnis, 2022). Networking and mentoring relationships will help her build the knowledge, skills, and social capital necessary to transition into the new job role. Emotional support also appears more frequently in our literature review as a resource for the retirement transition. At this point in her career, the pending career transition signifies a loss of her professional identity as she leaves the workforce. Emotional support for the retirement transition can include activities to engage the retiring woman in the community or other fulfilling aspects of life outside of the workplace.

Our data highlight instrumental support as an emphasis for career transitions to entrepreneurship, career re-entry, school-to-work transition, upward mobility, and the retirement transition. In the cases of each of these types of career transitions, the woman is entering unknown territory as she becomes her own boss, returns to the workforce after a substantial break, seeks to move from individual contributor to organizational leader, or leaves the workforce permanently. These changes require tangible resources to aid in a successful transition. For example, women transitioning to entrepreneurship need time and money to successfully start their business (Patterson and Mavin, 2009; Hodges, 2012; Duberley and Carrigan, 2013). In another case, a woman seeking career re-entry may require childcare to return to work (Greer, 2013; Arntz et al., 2017), especially if she was the primary caretaker of her children during the career break. The woman transitioning into retirement may benefit from instrumental support offered in the form of part-time work opportunities to help ease her career transition (Park et al., 2012; Zhan et al., 2015; Silver, 2016; Berg et al., 2017).

In our research, the higher frequencies of documented instrumental and emotional support for women's career transitions suggests these types of social support may be more common in successful career transitions among women than informational and appraisal support. Though informational and appraisal support appear to be documented less frequently in the career transition literature, these types of social support can still play an important role in career transitions for women. For example, the job-related knowledge and skills acquired through informational support are undoubtedly important in preparing a woman to pursue a new career opportunity (Greer, 2013; Greer and Minnis, 2022). Likewise, receiving positive feedback related to job performance and confidence boosting for the career transition process are examples of appraisal support that can contribute positively as a woman moves through a career transition by reducing feelings of low self-esteem and low self-efficacy (França, 2012; Lindstrom et al., 2012; Greer, 2013).

Making a successful career transition requires varied types of support for women who may face unfavorable conditions related to discrimination, financial insecurity, unfavorable work-life balance, and challenges to their identity (e.g., Fehring and Herring, 2012; Duberley and Carrigan, 2013; Greer, 2013; Yakaboski, 2016; Zimmerman and Clark, 2016). Accomplishing variety in their social networks may require women to recognize their need for different types of support and proactively seek them out.

#### Social Support and Support Providers

Based on our review of the literature and the proportions calculated in the resultant dataset (see Table 1), we found the *self* to be the most documented source of support for each type of career transition. The high frequencies of support attributed to the *self* could be an indication of the high value of self-efficacy, self-awareness, and self-management as coping resources during a career transition, which expands beyond the typical belief that women only need the right knowledge, skills, and work experiences to transition to a new job. We also found that instrumental and emotional support were often attributed to family, friends, and the career transitioner, herself. Therefore, our results may highlight the importance of personal connections and self-care in successful career transitions. Accordingly, women can nurture relationships and accept support from family and friends, while also being her own advocate for the new job opportunity. Although in our literature review, we found that most appraisal support was attributed to the *self*, women can also identify other people in their network who will provide this appraisal support during career transitions.

Women who are seeking a new profession or upward mobility might benefit from expanding their networks to include educators and trainers, the second most frequently documented source of social support for these two career transition types. According to our review, educators and trainers can provide informational support to expose women to the knowledge, skills, and experiences that prepare them for the career transition to a new profession or higher-level job. This additional informational support can help them acquire more knowledge about the roles and responsibilities associated with their new job position. Educators and trainers may be internal to the woman's current employer, or she may need to seek out educators and trainers in colleges, universities, professional societies, or adult and continuing education programs. In turn, the educators and trainers should give her the informational support needed to complete her desired career transition.

For women seeking a career transition to entrepreneurship or retirement, family and friends were the second most document source of support. Likewise, family and friends appear to be important for the school-to-work transition. Therefore, women can expand their networks to include family and friends as sources of social support during these types of career transitions. For the budding entrepreneur, family and friends can provide instrumental support, such as financial backing (Patterson and Mavin, 2009; Hodges, 2012; Duberley and Carrigan, 2013). This financial support can help these women overcome a major barrier to entrepreneurship. For women transitioning into retirement, family and friends can offer emotional support and keep the women engaged in the community through strong social bonds (Myers, 2011; Byles et al., 2013; Silver, 2016). This type of emotional support can help women transition into retirement by shifting their sense of purpose and identification to include their role in the community and their social systems. Similarly, for women transitioning from school to work, family and friends can offer emotional support to facilitate the experience of changing identities from student to worker (Mauro et al., 2016; Finn, 2017; Yuan and Ngai, 2018). Strong family relationships can be particularly supportive for women during this transition that generally marks the beginning of adulthood.

When attempting to transition back into the workforce following a career break, women could consider looking to potential employers/organizations to support the transition. During this transition, women have many needs for instrumental supports regarding their work conditions. Most notably, organizations and employers can provide a flexible work environment and family-friendly policies that promote work-life balance as women re-enter the workforce (Cabrera, 2009; Zimmerman and Clark, 2016; Arntz et al., 2017). The inability to achieve a satisfying work-life balance could deter women from seeking to re-enter the workforce.

### Limitations and Future Research

The results of our study contain insights for how women can be supported in transitioning to the next career experience. Recognizing the importance of context-based support during career transitions (Sun and Wang, 2009), we have clarified which types of social support are relevant for different types of career transitions while also identifying where the support tends to come from. Providers of social support can use this information to give women the types of support that can help them be successful in career transitions. However, we recognize that our results are limited to the chosen research methods, including the search terms we used to identify literature to include in this review. Though our study included 80 journal articles published over a 10-year period, it is possible that we may have arrived at different results using different search terms or different codification of the identified social support.

Furthermore, in our study, we noted all types and sources of support as documented in the literature. However, future research could empirically assess which support types and sources of support are actually used (not only theoretical) and most efficacious for women in different career transitions. This future research direction can overcome some of our limitations by incorporating additional research methods to include primary data collection and analysis that elucidates the nuances of social support for women in career transitions. Additional research is needed to determine the best timing for providing social support. For example, is emotional support more helpful before, during, or after the transition? Additionally, further research is needed to determine any relationships between the source of support, the types of support, and the *quality* of the career transition outcomes. The results of these future studies could help career development professionals and transitioners personalize their efforts for the nuanced experiences of women in specific career transitions. These future research avenues can contribute positively to women's career mobility, bolstering workforce diversity and women's career satisfaction.

## Data Availability Statement

The original contributions presented in the study are included in the article/Supplementary Material, further inquiries can be directed to the corresponding authors.

## Author Contributions

TG conceptualized the study, identified articles for the literature review, performed the quantitative data analysis, and contributed to writing the manuscript. AK performed the qualitative data analysis and contributed to writing the manuscript. Both authors contributed to the article and approved the submitted version.

## Conflict of Interest

The authors declare that the research was conducted in the absence of any commercial or financial relationships that could be construed as a potential conflict of interest.

## Publisher's Note

All claims expressed in this article are solely those of the authors and do not necessarily represent those of their affiliated organizations, or those of the publisher, the editors and the reviewers. Any product that may be evaluated in this article, or claim that may be made by its manufacturer, is not guaranteed or endorsed by the publisher.
