# Supplementary material for: Overcoming Barriers to Women's Career Transitions: A Systematic Review of Social Support Types and Providers
Source: Front Psychol. 2022 May 26;13:777110. doi: 10.3389/fpsyg.2022.777110 (PMC9204263; doi:10.3389/fpsyg.2022.777110)
Supplement: Supplementary file 1 [file Data_Sheet_1.docx]

# Tables

**Supplementary Table 1**

*Social Support Providers and Types of Support*

| **School-to-Work Transition - 32 articles** | | | | |
| --- | --- | --- | --- | --- |
| **Article Title** | | **Support Providers** | **Type of Support** | **Description** |
| The Bulgarian educational system and gender segregation in the labour market (Bieri et al., 2016) | | Organization/Employer | Instrumental | Gender equality |
| The Role of Parents and Peers in the Transition from University to Work Life (Buhl et al., 2018) | | Self | Instrumental | Career exploration |
|  |  | Family & Friends | Emotional | Strong social systems |
|  |  | Family & Friends | Emotional | Communication with significant other |
| Gendered Institutional Research Cultures in Science: The Post-doc Transition for Women Scientists (Case & Richley, 2013) | | Self | Appraisal | Self-awareness |
|  |  | Organization/Employer | Instrumental | Timely communication of decisions |
|  |  | Organization/Employer | Instrumental | Networking |
|  |  | Self | Instrumental | Publishing consistently |
|  |  | Mentors | Instrumental | Mentorships |
|  |  | Peers & Colleagues | Instrumental | Collaboration |
| Brown Suits Need Not Apply: The Intersection of Race, Gender, and class in institutional network building (Damaske, 2009) | | Education/Training | Informational | High academic standing |
|  |  | Self | Instrumental | Dressing professionally |
|  |  | Self | Emotional | Persistence |
| Closing the gaps and filling the STEM pipeline: a multidisciplinary approach (Doerschuk et al., 2016) | | Peers & Colleagues | Instrumental | Teamwork |
|  |  | Family & Friends | Emotional | Involvement in community |
|  |  | Peers & Colleagues | Emotional | Peer mentoring |
|  |  | Self | Instrumental | Career exploration |
|  |  | Self | Appraisal | Self-exploration |
|  |  | Self | Instrumental | Volunteering |
| Young Adult Couples Transitioning to Work: The Intersection of Career and Relationship (Domene et al., 2012) | Family & Friends | | Emotional | Communication with significant other |
|  | Friends & Family | | Emotional | Involvement in community |
|  | Family & Friends | | Instrumental | Cohabitation |
|  | Family & Friends | | Emotional | Strong relationship balance |
|  | Family & Friends | | Instrumental | Shared goals with significant other |
| Postgraduates’ perceptions of preparedness for work as a doctor and making future career decisions: Support for rural, non-traditional medical schools (Eley, 2010) | Educators & Trainers | | Informational | Extra learning experience |
|  | Organization/Employer | | Instrumental | Part-time work |
| Relational Transitions, Emotional Decisions: New Directions for Theorizing Graduate Employment (Finn, 2017) | Family & Friends | | Emotional | Strong social networks |
|  | Family & Friends | | Emotional | Strong familial relationships |
|  | Family & Friends | | Emotional | Living near family |
|  | Self | | Emotional | Communication |
|  | Family & Friends | | Emotional | Feeling valued |
| Advancing Our Legacy: a Black Feminist Perspective on the Significance of Mentoring for African-American Women in Educational Leadership (Grant, 2012) | Peers/Colleagues | | Instrumental | Peer mentoring |
| Gender-specific models of work-bound Korean adolescents' social supports and career adaptability on subsequent job satisfaction (Han & Rojewski, 2015) | Organization/Employer | | Instrumental | Career adaptability |
|  | Family & Friends | | Emotional | Strong social and familial relationships |
|  | Organization/Employer | | Informational | Awareness of resources |
|  | Self | | Instrumental | Proactive preparation |
| Critical moments in career construction counseling (Hartung & Vess, 2016) | Mentors | | Instrumental | Role models |
|  | Educators & Trainers | | Informational | Career counseling |
|  | Self | | Appraisal | Self-reflection |
|  | Self | | Appraisal | Setting goals |
| Persistent Disadvantages or New Opportunities? The Role of Agency and Structural Constraints for Low-Achieving Adolescents' School-to-Work Transitions (Holtmann et al., 2017) | Self | | Instrumental | Creating career plan |
|  | Educators & Trainers | | Informational | Decision-making resources |
|  | Educators & Trainers | | Informational | Diploma or certificate |
|  | Self | | Instrumental | Setting goals |
|  | Self | | Instrumental | Actively applying for jobs |
|  | Family & Friends | | Instrumental | Employed parents |
| Tolerance of Uncertainty: Links to Happenstance, Career Decision Self-efficacy, and Career Satisfaction (Kim et al., 2016) | Educators & Trainers | | Informational | Happenstance skills |
|  | Self | | Instrumental | Self-efficacy |
|  | Self | | Instrumental | Career satisfaction |
|  | Self | | Instrumental | Tolerance for uncertainty |
| Gender Matters in the Transition to Employment for Young Adults With Physical Disabilities (Lindsay et al., 2019) | Educators & Trainers | | Informational | Self-advocacy skills |
|  | Self | | Instrumental | Comfort disclosing disability |
|  | Educators & Trainers | | Informational | Condition management |
|  | Family & Friends | | Emotional | Strong social systems |
|  | Self | | Instrumental | Realistic expectations |
|  | Family & Friends | | Instrumental | Access to transportation |
| Gender gaps: Career development for young women with disabilities (Lindstrom et al., 2012) | Self | | Emotional | Confidence |
|  | Educators & Trainers | | Instrumental | Self-advocacy skills |
|  | Organization/Employer | | Informational | Access to career options |
|  | Self | | Instrumental | Broad perspective of options |
|  | Educators & Trainers | | Informational | Knowledgeable educators |
|  | Organization/Employer | | Instrumental | Accommodating workplaces |
|  | Self | | Informational | Awareness of disability |
| Gender Differences in Higher Education from a Life Course Perspective: Transitions and Social Inequality Between Enrolment and First Post-doc Position (Lörz & Mühleck, 2019) | Family & Friends | | Instrumental | Financial independence |
|  | Self | | Instrumental | Intrinsic motivation |
|  | Educators & Trainers | | Informational | High academic standing |
|  | Childcare Providers | | Instrumental | Access to childcare |
| Lost in Transition: College Resources and the Unequal Early-career Trajectories of Arts Alumni (Martin & Frenette, 2017) | Organization/Employer | | Instrumental | Flexible workplaces |
|  | Family & Friends | | Emotional | Involvement in community |
|  | Family & Friends | | Instrumental | Family connections to workplace |
|  | Organization/Employer | | Informational | Internships |
|  | Self | | Informational | Career development |
|  | Self | | Informational | Self-development |
|  | Family & Friends | | Emotional | Strong social systems |
| New Careers in Nursing Scholar Alumni Toolkit: An Innovative Resource for Transition to Practice (Mauro et al., 2016) | Mentors | | Instrumental | Mentorships |
|  | Self | | Instrumental | Realistic expectations |
|  | Family & Friends | | Emotional | Building social systems |
| Generation Y graduates and career transition: Perspectives by gender (Maxwell & Broadbridge, 2014) | Self | | Instrumental | Proactive planning |
|  | Self | | Instrumental | Actively looking for jobs |
|  | Self | | Instrumental | Realistic expectations |
| "I Am Haunted by the Question of What I Shall Do": the Vocational Struggle of a Teenage Girl in the 1940s as Seen Through Her Diary Account (Mills, 2017) | Family & Friends | | Emotional | Strong social systems |
|  | Mentors | | Instrumental | Role models and mentors |
|  | Self | | Appraisal | Self-reflection |
|  | Self | | Instrumental | Career exploration |
|  | Self | | Instrumental | Self-efficacy |
|  | Educators & Trainers | | Informational | High academic standing |
| The college-to-career transition: an exploration of emerging adulthood (Murphy et al., 2010) | Family & Friends | | Emotional | Strong social systems |
|  | Self | | Instrumental | Realistic expectations |
|  | Self | | Instrumental | Positive outlook |
|  | Organization/Employer | | Instrumental | Work-life balance |
|  | Family & Friends | | Instrumental | Financial security |
| Indigenous Fijian Female Pupils and Career Choice: Explaining Generational Gender Reproduction (Nilan, 2009) | Mentor | | Instrumental | Role models |
|  | Organization/Employer | | Informational | Awareness of career options |
|  | Educators & Trainers | | Informational | Gender equity training |
| School-to-work transition of career and technical education graduates (Packard, et al., 2012) | Educators & Trainers | | Informational | Job related to education |
|  | Educators & Trainers | | Informational | High academic standing |
|  | Self | | Instrumental | Proactive planning |
| A poisoned chalice? Why UK women engineering and technology students may receive more 'help' than their male peers (Powell et al., 2011) | Peers & Colleagues | | Instrumental | Collaboration with other students |
|  | Self | | Informational | Knowing how to conform in masculine culture |
| The Missing Women in STEM? Assessing Gender Differentials in the Factors Associated with the Transition to First Jobs (Sassler et al., 2017) | Educators & Trainers | | Informational | Early exposure to STEM |
|  | Self | | Instrumental | Self-efficacy |
|  | Self | | Instrumental | Positive outlook |
| Apprenticeships Should Work for Women Too! (Simon & Clarke, 2016) | Mentors | | Instrumental | Mentorships |
|  | Organization/Employer | | Informational | Access to career support |
|  | Educators & Trainers | | Informational | Job related to education |
|  | Mentors | | Instrumental | Role models |
|  | Family & Friends | | Emotional | Strong familial relationships |
| "Setting up for the Next Big Thing": Undergraduate Women Engineering Students Postbaccalaureate Career Decisions (Smith & Gayles, 2017) | Organization/Employer | | Informational | Internships |
|  | Family & Friends | | Emotional | Involvement in community |
|  | Organization/Employer | | Instrumental | Access to career support |
|  | Educators & Trainers | | Informational | Job related to education |
| The Role of Career Values for Work Engagement During the Transition to Working Life (Sortheix et al., 2013) | Self | | Instrumental | Positive mindset |
|  | Self | | Instrumental | Intrinsic motivation |
|  | Organization/Employer | | Emotional | Alignment between job and values |
| Women's Self-presentation and the Transition from Classroom to Workplace (Tazzyman, 2020) | Self | | Instrumental | Micro-resistance |
|  | Self | | Instrumental | Proactive planning |
| Sustaining the clinical and translational research workforce: Training and empowering the next generation of investigators (Yin et al., 2015) | Mentors | | Instrumental | Mentorships |
|  | Educators & Trainers | | Informational | Competency based training |
|  | Educators & Trainers | | Informational | Access to workshops and continued education |
| "My Youth, I Call the Shots": a Qualitative Examination of University Students' Perspectives on Life Transition and Identity Development in China (Yuan & Ngai, 2018) | Family & Friends | | Emotional | Involvement in community |
|  | Mentors | | Instrumental | Mentorships |
|  | Educators & Trainers | | Instrumental | Hands-on experience |
| Femininities and Masculinities in Highly Skilled Migration: Peruvian Graduates' Narratives of Employment Transitions and Binational Marriages in Switzerland (Seminario, 2018) | Family & Friends | | Emotional | Strong familial relationships |
|  | Organization/Employer | | Instrumental | Flexibility in job |
|  | Self | | Appraisal | Self-exploration |
| **Upward Mobility Transition - 12 articles** | | | | |
| **Article Title** | **Support Providers** | | **Type of Support** | **Description** |
| Looking through the glass ceiling: A qualitative study of STEM women’s career narratives (Amon, 2017) | Supervisors | | Appraisal | Recognition |
|  | Family & Friends | | Emotional | Strong social systems |
|  | Organization/Employer | | Instrumental | Work-life balance |
|  | Self | | Instrumental | Adaptive personality |
|  | Self | | Instrumental | Adaptive leadership styles |
|  | Peers & Colleagues | | Instrumental | Collaboration |
|  | Self | | Appraisal | Self-development |
| Leading a Quiet Revolution: Women High School Principals in Traditional Arab Society in Israel (Arar & Shapira, 2012) | Family & Friends | | Emotional | Strong familial relationships |
|  | Educators & Trainers | | Informational | Higher education |
|  | Educators & Trainers | | Instrumental | Leadership experience |
|  | Peers & Colleagues | | Instrumental | Teamwork |
|  | Peers & Colleagues | | Instrumental | Collaboration |
|  | Self | | Instrumental | Balancing identities |
|  | Self | | Instrumental | Communication |
| Barriers to the Executive Suite: Evidence from Ireland (Cross, 2010) | Supervisors | | Appraisal | Recognition |
|  | Organization/Employer | | Instrumental | Flexible workplace |
| The Gender Gap in Early Career Transitions in the Life Sciences (Lerchenmueller & Sorenson, 2018) | Mentors | | Instrumental | Role models |
|  | Self | | Instrumental | High productivity |
| Practitioner Application: Career Inflection Points of Women Who Successfully Achieved the Hospital CEO Position (Mastro, 2014) | Educators & Trainers | | Informational | Higher education |
|  | Mentors | | Instrumental | Mentoring |
|  | Organization/Employer | | Instrumental | Flexible workplace |
| Onto, up, off the Academic Faculty Ladder: The Gendered Effects of Family on Career Transitions for a Cohort of Social Science Ph.D.s (Morrison et al., 2011) | Self | | Instrumental | Drive |
|  | Educators & Trainers | | Informational | High academic standing |
|  | Self | | Instrumental | High productivity |
|  | Family & Friends | | Emotional | Support from older children |
| Moving up the Ladder: Heterogeneity Influencing Academic Career Through Research Orientation, Gender, and Mentors (Ooms et al., 2019) | Self | | Instrumental | High productivity |
|  | Self | | Informational | Integrating career and education |
|  | Mentors | | Instrumental | Mentoring |
| Analysis of postdoctoral training outcomes that broaden participation in science careers (Rybarczyk et al., 2016) | Educators & Trainers | | Informational | Quality training |
|  | Self | | Instrumental | High productivity |
|  | Self | | Instrumental | Proactive planning |
|  | Educators & Trainers | | Informational | Hands on experience |
|  | Mentors | | Instrumental | Mentoring |
|  | Self | | Instrumental | Competitive drive |
| Anticipatory guidance as a principle of faculty development: Managing transition and change (Schor et al, 2011) | Mentors | | Instrumental | Mentoring |
|  | Self | | Instrumental | Pushing boundaries of comfort zone |
|  | Organization/Employer | | Appraisal | Incentives |
| Leaks in the Pipeline: Separating Demographic Inertia from Ongoing Gender Differences in Academia (Shaw & Stanton, 2012) | Mentors | | Instrumental | Role models and mentors |
|  | Educators & Trainers | | Informational | Hands on experience |
|  | Self | | Instrumental | Drive |
|  | Family & Friends | | Emotional | Strong social systems |
| The transition to hospital consultant and the influence of preparedness, social support, and perception: A structural equation modelling approach (Westerman et al., 2013) | Family & Friends | | Emotional | Strong social systems |
|  | Supervisors | | Appraisal | Supervision from supportive superiors |
| Potential Parenthood and Career Progression of Men and Women -- a Simultaneous Hazards Approach (Biewen & Seifert, 2016) | Family & Friends | | Emotional | Cohabitation |
|  | Educators & Trainers | | Informational | Higher education |
| **Transition to a New Profession - 13 articles** | | | | |
| **Article Title** | **Support Providers** | | **Type of Support** | **Description** |
| Leaving the house: the experience of former members of parliament who left the house of commons in 2010 (Byrne & Theakston, 2016) | Self | | Instrumental | Maintaining involvement in field |
|  | Educators & Trainers | | Informational | Building job search skills |
| Career development for women veterans: Facilitating successful transitions from military service to civilian employment (Greer, 2017) | Self | | Appraisal | Changing the situation |
|  | Self | | Emotional | Coping strategies |
|  | Self | | Emotional | Stress management |
|  | Family & Friends | | Emotional | Sharing experiences |
| Are Career Termination Concerns Only for Athletes? A Case Study of the Career Termination of an Elite Female Coach (Kenttä & Mellalieu, 2016) | Family & Friends | | Emotional | Fostering relationships |
|  | Family & Friends | | Instrumental | Building support networks |
|  | Self | | Appraisal | Self-reflection |
|  | Self | | Informational | Career exploration |
|  | Organization/Employer | | Instrumental | Perceived or received career resources |
|  | Self | | Emotional | Coping strategies |
| Adults changing careers through university education: making meaning of quantitative career assessment scores through an integrative structured interview (McMahon et al., 2018) | Self | | Appraisal | Self-reflection |
|  | Self | | Appraisal | Career transition reflection |
|  | Self | | Emotional | Self-exploration |
| Relational processes in career transition: extending theory, research, and practice (Motulsky, 2010) | Family & Friends | | Emotional | Strong familial relationships |
|  | Peers & Colleagues | | Emotional | Strong peer relationships |
|  | Mentors | | Emotional | Strong mentoring relationships |
|  | Organization/Employer | | Instrumental | Challenging career |
|  | Organization/Employer | | Emotional | Supportive work environment |
|  | Educators & Trainers | | Informational | Therapy |
|  | Family & Friends | | Emotional | Owning pets |
| A longitudinal qualitative exploration of elite Korean tennis players' career transition experiences (Park et al., 2013) | Self | | Instrumental | Building new identity |
|  | Self | | Informational | Early transition preparation |
|  | Self | | Emotional | Coping strategies |
|  | Family & Friends | | Emotional | Strong social system |
|  | Educators & Trainers | | Informational | Developing transferrable skills |
|  | Self | | Informational | Lifestyle exploration |
|  | Self | | Instrumental | Perceived or actual control over transition decision |
| Examining the Long-term Impact of Participating in a Professional Development Community of Music Teacher Educators in the USA: an Anchor Through Turbulent Transitions (Pellegrino et al., 2018) | Peers & Colleagues | | Emotional | Strong peer relationships |
|  | Mentors | | Emotional | Strong mentoring relationships |
|  | Family & Friends | | Emotional | Authentic community |
|  | Peers & Colleagues | | Instrumental | Engaging in collaborative projects |
| STEM Career changers' transformation into science teachers (Snyder et al., 2013) | Peers & Colleagues | | Informational | Observation of experienced co-workers |
|  | Peers & Colleagues | | Instrumental | Engaging in collaborative projects |
|  | Mentors | | Instrumental | Identifying role models |
|  | Self | | Instrumental | Taking ownership of work environment |
| The relationship between dual-career and post-sport career transition among elite athletes in South Africa, Botswana, Namibia, and Zimbabwe (Tshube & Feltz, 2015) | Family & Friends | | Emotional | Strong social system |
|  | Organization/Employer | | Instrumental | Working part-time |
|  | Self | | Instrumental | Perceived or actual control over transition decision |
|  | Educators & Trainers | | Informational | Developing transferrable skills |
| Personal strategies for managing a second career: the experiences of Spanish Olympians (Vilanova & Puig, 2016) | Educators & Trainers | | Informational | Education |
|  | Educators & Trainers | | Informational | Work experience |
|  | Organization/Employer | | Instrumental | Networking |
|  | Educators & Trainers | | Informational | Developing transferrable skills |
|  | Self | | Instrumental | Financial security |
| Proven Leadership = College Credit: Enhancing Employability of Transitioning Military Members Through Prior Learning Assessment (Bergman & Herd, 2017) | Organization/Employer | | Instrumental | Legislation (Post-9/11 GI Bill) |
|  | Educators & Trainers | | Informational | Education (Prior Learning Assessment) |
| From the Pulpits to the Boards: A Study on Prospective Second Career Teachers in Turkey (Unisen & Polat, 2016) | Self | | Emotional | Respect for others’ beliefs |
|  | Self | | Appraisal | Adapting self to engage and involve others |
|  | Educators & Trainers | | Instrumental | Creating challenging teaching materials |
|  | Educators & Trainers | | Informational | Communication skills |
| Reframing the Two-body Problem in U.S. STEM Departments: Asian Women Faculty Negotiation of Career and Family (Yokaboski, 2016) | Organization/Employer | | Emotional | Supportive organization/employer |
|  | Family & Friends | | Emotional | Strong familial relationships |
| **Transition to Entrepreneurship - 5 articles** | | | | |
| **Article Title** | **Support Providers** | | **Type of Support** | **Description** |
| The career identities of ‘mumpreneurs’: Women’s experiences of combining enterprise and motherhood (Duberley & Carrigan, 2013) | Self | | Emotional | Pride in self-management |
|  | Self | | Instrumental | Developing identity |
|  | Family & Friends | | Instrumental | Financial security (due to spouse’s income) |
|  | Childcare Providers | | Instrumental | Access to childcare |
| The transition of midlife women from organizational employment to self-employment (Hodges, 2012) | Self | | Appraisal | Positive perception |
|  | Self | | Appraisal | Measuring success in terms of fulfillment |
|  | Self | | Emotional | Confidence |
|  | Family & Friends | | Instrumental | Financial security (due to spouse’s income) |
|  | Self | | Instrumental | Setting personal goals |
| The Entrepreneurship-motherhood Nexus: A longitudinal investigation from a boundaryless career perspective (Lewis et al., 2015) | Self | | Instrumental | Creating boundaries |
|  | Self | | Instrumental | Developing identity |
|  | Educators & Trainers | | Informational | Transferrable skills |
|  | Childcare Providers | | Instrumental | Childcare |
|  | Family & Friends | | Emotional | Strong familial relationships |
| Women Entrepreneurs: Jumping the Corporate Ship and Gaining New Wings (Patterson & Mavin, 2009) | Family & Friends | | Instrumental | Financial security (due to spouse’s income) |
|  | Self | | Emotional | Confidence |
|  | Family & Friends | | Emotional | Strong familial relationships |
| The Role of Developmental Relationships in the Transition to Entrepreneurship: A Qualitative Study and Agenda for Future Research (Terjesen & Sullivan, 2011) | Mentors | | Instrumental | Mentorships |
|  | Mentors | | Emotional | Maintaining mentorship from corporate job |
|  | Self | | Appraisal | Awareness of reality of changes |
|  | Mentors | | Informational | Technological support in locating mentors |
|  | Educators & Trainers | | Informational | Education |
| **Career Re-entry Transition - 9 articles** | | | | |
| **Article Title** | **Support Providers** | | **Type of Support** | **Description** |
| The Sorting of Female Careers After First Birth: A Competing Risks Analysis of Maternity Leave Duration (Arntz et al., 2017) | Self | | Instrumental | Financial security |
|  | Organization/Employer | | Instrumental | Regular hours |
|  | Organization/Employer | | Instrumental | Tenure |
|  | Organization/Employer | | Instrumental | Work-family balance |
|  | Childcare Providers | | Instrumental | Access to childcare |
| Protean Organizations: Reshaping Work & Careers to Retain Female Talent (Cabrera, 2009) | Organization/Employer | | Instrumental | Flexible work environment |
|  | Self | | Instrumental | Self-direction |
|  | Self | | Instrumental | Proactive planning |
| A Career and Learning Transitional Model for Those Experiencing Labour Market Disadvantage (Cameron, 2009) | Self | | Emotional | Focus on the present |
|  | Self | | Appraisal | Focus on self-concept |
| Voices from the "working Lives" Project: The Push-pull of Work and Care (Fehring & Herring, 2012) | Self | | Emotional | Acceptance of transition |
|  | Self | | Instrumental | Self-employment |
| Women and Labor Market: Work Family Conflict and Career Self-management (França, 2012) | Family & Friends | | Emotional | Strong social systems |
|  | Self | | Instrumental | High self-efficacy |
| Facilitating Successful Re-Entries in the United States: Training and Development for Women Returners (Greer, 2013) | Self | | Emotional | Confidence |
|  | Educators & Trainers | | Informational | Training |
|  | Family & Friends | | Emotional | Strong familial support |
|  | Childcare Providers | | Instrumental | Access to childcare |
|  | Organization/Employer | | Instrumental | Networking |
|  | Self | | Informational | Knowledge of job market |
|  | Organization/Employer | | Instrumental | Part-time work |
|  | Self | | Instrumental | Proactive planning |
| Achievement goals and autonomy: How person−context interactions predict effective functioning and well-being during a career transition (Heidemeier & Wiese, 2014) | Organization/Employer | | Instrumental | Work autonomy |
|  | Self | | Instrumental | Setting goals |
|  | Organization/Employer | | Emotional | Returning to same organization/employer |
| A Multilevel Perspective of the Identity Transition to Motherhood (Hennekam et al., 2019) | Family & Friends | | Emotional | Strong social systems |
|  | Organization/Employer | | Emotional | Family-friendly organization/employers |
|  | Mentors | | Instrumental | Identifying role models |
|  | Family & Friends | | Emotional | Strong familial systems |
| Opting-Out and Opting-In: A Review and Agenda for Future Research (Zimmerman & Clark, 2016) | Self | | Instrumental | Proactive planning |
|  | Self | | Appraisal | Focus on self-concept |
|  | Self | | Instrumental | Maintaining flexibility |
|  | Organization/Employer | | Instrumental | Flexible work environment |
| **Transition to Retirement - 9 articles** | | | | |
| **Article Title** | **Support Providers** | | **Type of Support** | **Description** |
| Insights Into Life After Sport for Spanish Olympians: Gender and Career Path Perspectives (Barriopedro et al., 2018) | Organization/Employer | | Instrumental | Part-time work during transition |
|  | Self | | Instrumental | Proactive planning |
| The Relationship Between Employer‐provided Training and the Retention of Older Workers: Evidence from Germany (Berg, et al., 2017) | Organization/Employer | | Instrumental | Part-time work during transition |
|  | Educators & Trainers | | Informational | Training |
|  | Organization/Employer | | Instrumental | Higher wages |
| Transforming Retirement: New Definitions of Life After Work (Byles et al., 2013) | Self | | Emotional | Satisfaction with career |
|  | Family & Friends | | Emotional | Engagement in community |
|  | Self | | Appraisal | Self-exploration |
|  | Self | | Appraisal | Positive view of retirement |
| Experiences of the Pre- and Post-retirement Period of Female Elite Artistic Gymnasts: An Exploratory Study (Clowes et al., 2015) | Self | | Emotional | Keeping in touch with Organization/Employer |
|  | Organization/Employer | | Instrumental | Transitioning to new role |
|  | Family & Friends | | Emotional | Engagement in community |
|  | Family & Friends | | Emotional | Building social systems |
| Career Pathways into Retirement in the UK: Linking Older Women’s Pasts to the Present (Duberley & Carmichael, 2016) | Self | | Appraisal | Positive view of retirement |
|  | Organization/Employer | | Instrumental | Protean career before transition |
|  | Educators & Trainers | | Informational | Obtaining education |
|  | Self | | Instrumental | Proactive planning |
| Self-initiated Expatriation (SIE) in Older Women: Exploring a Different Terrain (Myers, 2011) | Family & Friends | | Emotional | Engagement in community |
|  | Organization/Employer | | Instrumental | Part-time work |
|  | Family & Friends | | Emotional | Building social systems |
|  | Self | | Appraisal | Positive view of retirement |
| Exploring the retirement from sport decision-making process based on the transtheoretical model (Park et al., 2012) | Self | | Instrumental | Proactive planning |
|  | Self | | Emotional | Confidence |
|  | Family & Friends | | Emotional | Strong social systems |
|  | Educators & Trainers | | Informational | Building transferrable skills |
|  | Organization/Employer | | Instrumental | Part-time work |
|  | Self | | Appraisal | Self-exploration |
| An inquiry into self-Identification with retirement (Silver, 2016) | Family & Friends | | Emotional | Engagement in community |
|  | Family & Friends | | Emotional | Engaging in satisfying activities |
|  | Self | | Appraisal | Self-exploration |
|  | Organization/Employer | | Instrumental | Part-time work |
|  | Self | | Instrumental | Proactive planning |
| Retirees' motivational orientations and bridge employment: testing the moderating role of gender (Zhan et al., 2015) | Family & Friends | | Emotional | Engagement in community |
|  | Family & Friends | | Emotional | Engaging in family life |
|  | Organization/Employer | | Instrumental | Part-time work |

# References

Amon, M. J. (2017). Looking through the glass ceiling: A qualitative study of STEM women’s career narratives. *Frontiers in Psychology, 8*, 1-10. <https://doi.org/10.3389/fpsyg.2017.00236>

Arar, K. & Shapira, T. (2012). Leading a quiet revolution: Women high school principals in traditional Arab society in Israel. *Journal of School Leadership, 22*, 853-874. <https://doi.org/10.1177%2F105268461202200502>

Arntz, M., Dlugosz, S., & Wilke, R. A. (2017). The sorting of female careers after first birth: A competing risks analysis of maternity leave duration. *Oxford Bulletin of Economics and Statistics, 79*(5), 689-716. <https://doi.org/10.1111/obes.12158>

Barriopedro, M., Lopez de Subijana, C., & Muniesa C. (2018). Insights into life after sport for Spanish Olympians: Gender and career path perspectives. *Plos One, 13*(12). <https://doi.org/10.1371/journal.pone.0209433>

Berg, P. B., Hamman, M. K., Piszczek, M. M., & Ruhm, C. J. (2017). The relationship between employer‐provided training and the retention of older workers: Evidence from Germany. *International Labour Review*, *156*(3-4), 495-523. <https://doi.org/10.1111/ilr.12031>

Bergman, M. & Herd, A. (2017). Proven leadership = college credit: Enhancing employability of transitioning military members through prior learning assessment. *Advances in Developing Human Resources, 19*(1), 78-87. <https://doi.org/10.1177%2F1523422316682949>

Bieri, F., Imdorf, C., Stoilova, R., & Boyadjieva, P. (2016). The Bulgarian educational system and gender segregation in the labour market. *European Societies*, *18*(2), 158-179. <https://doi.org/10.1080/14616696.2016.1141305>

Biewen, M., & Seifert, S. (2018). Potential parenthood and career progression of men and women–a simultaneous hazards approach. *The BE Journal of Economic Analysis & Policy*, *18*(2), 20170154. <https://doi.org/10.1515/bejeap-2017-0154>

Buhl, H. M., Noack, P., & Kracke, B. (2018). The role of parents and peers in the transition from university to work life. *Journal of Career Development*, *45*(6), 523-535. <https://doi.org/10.1177%2F0894845317720728>

Byles, J., Tavener, M., Robinson, I., Parkinson, L., Smith, P. W., Stevenson, D., Leigh, L., Curryer, C. (2013). Transforming retirement: New definitions of life after work. *Journal of Women & Aging, 25*(1), 24-44. <https://doi.org/10.1080/08952841.2012.717855>

Byrne, C. & Theakston, K. (2016). Leaving the House: The experience of former members of Parliament who left the House of Commons in 2010. *Parliamentary Affairs, 69*(3), 686-707. <https://doi.org/10.1093/pa/gsv053>

Cabrera, E. F. (2009). Protean organizations: Reshaping work and careers to retain female talent. The Career Development International, 14(2), 186–201. <https://doi.org/10.1108/13620430910950773>

Cameron, R. (2009). A career and learning transitional model for those experiencing labour market disadvantage. *Australian Journal of Career Development, 18*(1), 17-25. <https://doi.org/10.1177%2F103841620901800104>

Case, S. S., & Richley, B. A. (2013). Gendered institutional research cultures in science: The post-doc transition for women scientists. *Community, Work & Family*, *16*(3), 327-349. <https://doi.org/10.1080/13668803.2013.820097>

Clowes, H., Lindsay, P., Fawcett, L., & Knowles, Z. R. (2015). Experiences of the pre and post retirement period of female elite artistic gymnasts: An exploratory study. *Sport & Exercise Psychology Review, 11*(2), 1-35. <https://researchonline.ljmu.ac.uk/id/eprint/1210>

Cross, C. (2010). Barriers to the executive suite: Evidence from Ireland. *Leadership & Organization Development Journal, 31*(2), 104-119. <https://doi.org/10.1108/01437731011024376>

Damaske, S. (2009, June). Brown suits need not apply: The intersection of race, gender, and class in institutional network building 1. In *Sociological Forum* (Vol. 24, No. 2, pp. 402-424). Oxford, UK: Blackwell Publishing Ltd. <https://doi.org/10.1111/j.1573-7861.2009.01105.x>

Doerschuk, P., Bahrim, C., Daniel, J., Kruger, J., Mann, J., & Martin, C. (2016). Closing the gaps and filling the STEM pipeline: A multidisciplinary approach. *Journal of Science Education and Technology*, *25*(4), 682-695. <https://doi.org/10.1007/s10956-016-9622-8>

Domene, J. F., Nee, J. J., Cavanaugh, A. K., McLelland, S., Stewart, B., Stephenson, M., Kauffman, B., Tse, C. K., Young, R. A. (2012). Young adult couples transitioning to work: The intersection of career and relationship. *Journal of Vocational Behavior, 81*(1), 17-25. <https://doi.org/10.1016/j.jvb.2012.03.005>

Duberley, J. & Carmichael, F. (2016). Career pathways into retirement in the UK: Linking older women’s pasts to the present. *Gender, Work, & Organization, 23*(6), 582-599. <https://doi.org/10.1111/gwao.12144>

Duberley, J. & Carrigan, M. (2013). The career identities of ‘mumpreneurs’: Women’s experiences of combining enterprise and motherhood. *International Small Business Journal: Researching Entrepreneurship, 31*(6), 629-651. <https://doi.org/10.1177%2F0266242611435182>

Eley, D. S. (2010). Postgraduates’ perceptions of preparedness for work as a doctor and making future career decisions: Support for rural, non-traditional medical schools. *Education for Health, 23*(2), 374-385. <https://www.educationforhealth.net/text.asp?2010/23/2/374/101489>

Fehring, H. & Herring, K. (2012). Voices from the Working Lives Project: The push-pull of work and care. *International Education Studies, 5*(6), 204-218. <https://eric.ed.gov/?id=EJ1067046>

Finn, K. (2017). Relational transitions, emotional decisions: New directions for theorising graduate employment. *Journal of Education and Work*, *30*(4), 419-431. <https://doi.org/10.1080/13639080.2016.1239348>

França, T. (2012). Women and labor market: Work family conflict and career self-management. Pensamento & Realidade, 27(4), 51-70. <https://revistas.pucsp.br/pensamentorealidade/article/view/14725>

Grant, C. M. (2012). Advancing our legacy: A Black feminist perspective on the significance of mentoring for African-American women in educational leadership. *International Journal of Qualitative Studies in Education*, *25*(1), 101-117. <https://doi.org/10.1080/09518398.2011.647719>

Greer, T. W. (2013). Facilitating successful re-entries in the United States: Training and development for women returners. *New Horizons in Adult Education & Human Resource Development, 25*(3), 41-61. <https://doi.org/10.1002/nha3.20030>

Greer, T. W. (2017). Career development for women veterans: Facilitating successful transitions from military service to civilian employment. *Advances in Developing Human Resources, 19*(1), 54-65. <https://doi.org/10.1177%2F1523422316682737>

Han, H., & Rojewski, J. W. (2015). Gender-specific models of work-bound Korean adolescents’ social supports and career adaptability on subsequent job satisfaction. *Journal of Career Development*, *42*(2), 149-164. <https://doi.org/10.1177%2F0894845314545786>

Hartung, P. J., & Vess, L. (2016). Critical moments in career construction counseling. *Journal of Vocational Behavior*, *97*, 31-39. <https://doi.org/10.1016/j.jvb.2016.07.014>

Heidemeier, H. & Wiese, B. S. (2014). Achievement goals and autonomy: How person-context interactions predict eddective dunctioning and well-being during a career transition. *Journal of Occupational Health Psychology, 19*(1), 18-31. <https://psycnet.apa.org/doi/10.1037/a0034929>

Hennekam, S., Syed, J., Faiza, A., & Dumazert, J. (2019). A multilevel perspective of the identity transition to motherhood. *Gender, Work, & Organization, 26*(7), 915-933. <https://doi.org/10.1111/gwao.12334>

Hodges, J. (2012). The transition of midlife women from organizational into self-employment. *Gender in Management: An International Journal, 27*(3), 186-201. <https://doi.org/10.1108/17542411211221277>

Holtmann, A. C., Menze, L., & Solga, H. (2017). Persistent disadvantages or new opportunities? The role of agency and structural constraints for low-achieving adolescents’ school-to-work transitions. *Journal of Youth and Adolescence*, *46*(10), 2091-2113. <https://doi.org/10.1007/s10964-017-0719-z>

Kenttä, G., Mellalieu, S., & Roberts, C. M. (2016). Are career termination concerns only for athletes? A case study of the career termination of an elite female coach. *The Sport Psychologist*, *30*(4), 314-326. <https://doi.org/10.1123/tsp.2015-0134>

Lerchenmueller, M. J. & Sorenson, O. (2018). The gender gap in early career transitions in the life sciences. *Research Policy, 47,* 1007-1017. <https://doi.org/10.1016/j.respol.2018.02.009>

Lewis, K. V., Harris, C., Morrison, R., & Ho, M. (2015). The entrepreneurship-motherhood nexus. *Career Development International, 20*(1), 21-37. <https://doi.org/10.1108/CDI-07-2014-0090>

Lindsay, S., Cagliostro, E., Albarico, M., Mortaji, N., & Srikanthan, D. (2019). Gender matters in the transition to employment for young adults with physical disabilities. *Disability and Rehabilitation*, *41*(3), 319-332. <https://doi.org/10.1080/09638288.2017.1390613>

Lindstrom, L., Harwick, R. M., Poppen, M., & Doren, B. (2012). Gender gaps: Career development for young women with disabilities. *Career Development and Transition for Exceptional Individuals*, *35*(2), 108-117. <https://doi.org/10.1177%2F0885728812437737>

Lörz, M., & Mühleck, K. (2019). Gender differences in higher education from a life course perspective: Transitions and social inequality between enrolment and first post-doc position. *Higher Education*, *77*(3), 381-402. <https://doi.org/10.1007/s10734-018-0273-y>

Martin, N. D. & Frenette, A. (2017). Lost in transition: College resources and the unequal early-career trajectories of Arts alumni. *American Behavioral Scientist, 61*(12), 1487-1509. <https://doi.org/10.1177%2F0002764217734273>

Mastro, M. L. (2014). Practitioner application: Career inflection points of women who successfully achieved the hospital CEO position. *Journal of Healthcare Management*, *59*(5), 383-384. <https://journals.lww.com/jhmonline/Citation/2014/09000/PRACTITIONER_APPLICATION__Career_Inflection_Points.12.aspx>

Mauro, A. M., Escallier, L. A., & Rosario-Sim, M. G. (2016). New careers in nursing scholar alumni toolkit: Development of an innovative resource for transition to practice. *Journal of Professional Nursing: Official Journal of the American Association of Colleges of Nursing*, *32*(5S), S59–S62. <https://doi.org/10.1016/j.profnurs.2016.02.002>

Maxwell, G. A. & Broadbridge, A. (2014). Generation Y graduates and career transition: Perspectives by gender. *European Management Journal, 32*(4), 547-553. <https://doi.org/10.1016/j.emj.2013.12.002>

McMahon, M., Watson, M., & Zietsman, L. (2018). Adults changing careers through university education: Making meaning of quantitative career assessment scores through an integrative structured interview. *South African Journal of Industrial Psychology, 44*(4), 1-10. <https://hdl.handle.net/10520/EJC-ee977d534>

Mills, R. (2017). “I am haunted by the question of what I shall do”: The vocational struggles of a teenage girl in the 1940s as seen through her diary accounts. *Journal of the Indiana Academy of the Social Sciences, 17*(1), 71-96. <https://digitalcommons.butler.edu/jiass/vol17/iss1/7>

Morrison, E., Rudd, E., & Nerad, M. (2011). Onto, up, off the academic faculty ladder: The gendered effects of family on career transitions for a cohort of social science Ph. Ds. *The Review of Higher Education*, *34*(4), 525-553. <https://doi.org/10.1353/rhe.2011.0017>

Motulsky, S. L. (2010). Relational processes in career transition: Extending theory, research, and practice. *The Counseling Psychologist*, *38*(8), 1078-1114. <https://doi.org/10.1177%2F0011000010376415>

Murphy, K. A., Blustein, D. L., Bohlig, A. J., & Platt, M. G. (2010). The college-to-career transition: An exploration of emerging adulthood. *Journal of Counseling and Development, 88*(2), 174-181. <https://doi.org/10.1002/j.1556-6678.2010.tb00006.x>

Myers, B. (2011). Self-Initiated Expatriation (SIE) in older women: Exploring a different terrain. *Women’s Studies Journal, 25*(2), 101-106. <http://www.wsanz.org.nz/journal/docs/WSJNZ252Myers101-106.pdf>

Nilan, P. (2009). Indigenous Fijian female pupils and career choice: Explaining generational gender reproduction. *Asia Pacific Journal of Education*, *29*(1), 29-43. <https://doi.org/10.1080/02188790802655031>

Ooms, W., Werker, C., & Hopp, C. (2019). Moving up the ladder: Heterogeneity influencing academic careers through research orientation, gender, and mentors. *Studies in Higher Education*, *44*(7), 1268-1289. <https://doi.org/10.1080/03075079.2018.1434617>

Packard, B. W. L., Leach, M., Ruiz, Y., Nelson, C., & DiCocco, H. (2012). School‐to‐work transition of career and technical education graduates. *The Career Development Quarterly*, *60*(2), 134-144. <https://doi.org/10.1002/j.2161-0045.2012.00011.x>

Park, S., Tod, D., & Lavallee, D. (2012). Exploring the retirement from sport decision-making process based on the transtheoretical model. *Psychology of Sport and Exercise*, *13*(4), 444-453. <https://doi.org/10.1016/j.psychsport.2012.02.003>

Park, S., Lavallee, D., & Tod, D. (2013). A longitudinal qualitative exploration of elite Korean tennis players’ career transition experiences. *Athletic Insight*, *15*(1), 65-92. <http://www.athleticinsight.com/Vol15Iss1/Transition.htm>

Patterson, N. & Mavin, S. (2009). Women entrepreneurs: Jumping the corporate ship and gaining new wings. *International Small Business Journal: Researching Entrepreneurship, 27*(2), 173-192. <https://doi.org/10.1177%2F0266242608100489>

Pellegrino, K., Kastner, J. D., Reese, J., & Russell, H. A. (2018). Examining the long-term impact of participating in a professional development community of music teacher educators in the USA: An anchor through turbulent transitions. *International Journal of Music Education, 36*(2), 145-159. <https://doi.org/10.1177%2F0255761417704214>

Powell, A., Dainty, A., & Bagilhole, B. (2011). A poisoned chalice? Why UK women engineering and technology students may receive more ‘help’ than their male peers. *Gender and Education*, *23*(5), 585-599. <https://doi.org/10.1080/09540253.2010.527826>

Rybarczyk, B. J., Lerea, L., Whittington, D., & Dykstra, L. (2016). Analysis of postdoctoral training outcomes that broaden participation in science careers. *Life Science Education, 15*(3), 1-11. <https://doi.org/10.1187/cbe.16-01-0032>

Sassler, S., Glass, J., Levitte, Y., & Michelmore, K. M. (2017). The missing women in STEM? Assessing gender differentials in the factors associated with transition to first jobs. *Social Science Research*, *63*, 192-208. <https://doi.org/10.1016/j.ssresearch.2016.09.014>

Schor, N. F., Guillet, R., & McAnarney, E. R. (2011) Anticipatory guidance as a principle of faculty development: Managing transition and change. *Academic Medicine, 86*(10), 1235-1240 <https://doi.org/10.1097/ACM.0b013e31822c1317>

Seminario, R. (2018). Femininities and masculinities in highly skilled migration: Peruvian graduates’ narratives of employment transitions and binational marriages in Switzerland. *Migration Letters*, *15*(1), 85-98. <https://doi.org/10.33182/ml.v15i1.338>

Shaw, A. K. & Stanton, D. E. (2012). Leaks in the pipeline: Separating demographic inertia from ongoing gender differences in academia. *Proceedings of the Royal Society, 279*(1743), 3736-3741. <https://doi.org/10.1098/rspb.2012.0822>

Silver, M. P. (2016). An inquiry into self-identification with retirement. *Journal of Women & Aging*, *28*(6), 477-488. <https://doi.org/10.1080/08952841.2015.1018068>

Simon, L., & Clarke, K. (2016). Apprenticeships should work for women too!. *Education + Training*, 58(6), 578-596. <https://doi.org/10.1108/ET-02-2016-0022>

Smith, K. N., & Gayles, J. G. (2017). "Setting up for the next big thing": Undergraduate women engineering students' postbaccalaureate career decisions. *Journal of College Student Development*, *58*(8), 1201-1217. <https://doi.org/10.1353/csd.2017.0094>

Snyder, C., Oliveira, A. W., & Paska, L. M. (2013). STEM Career Changers’ transformation into science teachers. *Journal of Science Teacher Education, 24*, 617-644. <https://doi.org/10.1007/s10972-012-9325-9>

Sortheix, F. M., Dietrich, J., Chow, A., & Salmela-Aro, K. (2013). The role of career values for work engagement during the transition to working life. *Journal of Vocational Behavior*, *83*(3), 466-475. <https://doi.org/10.1016/j.jvb.2013.07.003>

Tazzyman, A. (2020). Women's self‐presentation and the transition from classroom to workplace. *Gender, Work & Organization*, *27*(3), 327-346. <https://doi.org/10.1111/gwao.12375>

Terjesen, S., & Sullivan, S. E. (2011). The role of developmental relationships in the transition to entrepreneurship: A qualitative study and agenda for future research. *Career Development International, 16*(5), 482-506. <https://doi.org/10.1108/13620431111168895>

Tshube, T. & Feltz, D. (2015). The relationship between dual-career and post-sport career transition among elite athletes in South Africa, Botswana, Namibia and Zimbabwe. *Psychology of Sport and Exercise, 21,* 109-114. <https://doi.org/10.1016/j.psychsport.2015.05.005>

Unisen, A., & Polat, H. (2016). From the pulpits to the boards: A study on prospective second career teachers in Turkey. *International Education Studies*, *9*(9), 170-181. <https://doi.org/10.5539/ies.v9n9p170>

Vilanova, A., & Puig, N. (2016). Personal strategies for managing a second career: The experiences of Spanish Olympians. *International Review for the Sociology of Sport*, *51*(5), 529-546. <https://doi.org/10.1177%2F1012690214536168>

Westerman, M., Teunissen, P. W., Fokkema, J. P. I., van der Vleuten, C. P. M., Scherpbier, A. J.J.A., Siegert, C. E. H., Scheele, F. (2013). The transition to hospital consultant and the influence of preparedness, social support, and perception: A structural equation modelling approach. *Medical Teacher, 35*(4), 320-327. <https://doi.org/10.3109/0142159X.2012.735381>

Yin, H. L., Gabrilove, J., Jackson, R., Sweeney, C., Fair, A. M., & Toto, R. (2015). Sustaining the clinical translational research workforce: training and empowering the next generation of investigators. *Academic medicine: Journal of the Association of American Medical Colleges*, *90*(7), 861-865. <https://dx.doi.org/10.1097%2FACM.0000000000000758>

Yuan, R., & Ngai, S. S. Y. (2018). “My youth, I call the shots”: A qualitative examination of university students’ perspectives on life transition and identity development in China. *Children and Youth Services Review*, *94*, 140-147. <https://doi.org/10.1016/j.childyouth.2018.09.042>

Zhan, Y., Wang, M., & Shi, J. (2015). Retirees’ motivational orientations and bridge employment: Testing the moderating role of gender. *Journal of Applied Psychology*, *100*(5), 1319-1331. <https://psycnet.apa.org/doi/10.1037/a0038731>

Zimmerman, L. M. & Clark, M. A. (2016). Opting-out and opting-in: A review and agenda for future research. *Career Development International, 21*(6), 603-633. <https://doi.org/10.1108/CDI-10-2015-0137>
